# Partial purification and characterization of a broad‐spectrum bacteriocin produced by a *Lactobacillus plantarum* zrx03 isolated from infant's feces

**DOI:** 10.1002/fsn3.1428

**Published:** 2020-03-18

**Authors:** Shuang Lei, Ruixiang Zhao, Junliang Sun, Junjian Ran, Xiaoli Ruan, Yang Zhu

**Affiliations:** ^1^ School of Food Science Henan Institute of Science and Technology Xinxiang China; ^2^ Wageningen University and Research Centre Wageningen The Netherlands

**Keywords:** antimicrobial stability, bacteriocin, *Lactobacillus plantarum* zrx03, purification

## Abstract

*Lactobacillus plantarum* zrx03 was a bacteriocin‐producing strain isolated from infant's feces. The fermentation supernatant produced by this strain could strongly inhibit *Escherichia coli* JM109 ATCC 67387, *Staphylococcus aureus* ATCC 25923, and *Listeria monocytogenes* CICC 21633, in which the diameter of inhibition zone was 12.83 ± 0.62 mm, 15.08 ± 0.31 mm, 6.75 ± 0.20 mm, respectively, compared with lactic acid bacteria N1, N2, M13, M21, M31, and M37. According to amplification of 16S rRNA gene and identification of phylogenetic tree, this strain had a 1,450 bp sequence and 100% identity to the *L. plantarum* strain. Based on the influence of different protease treatments, such as pepsin, trypsin, papain, and proteinase K on the antimicrobial activity, this antimicrobial substance was considered to be a natural protein. Using bacteriocin produced by this strain as study object of this experiment, it had been extracted from ammonium sulfate precipitation and different organic solvents. The results showed that ethyl acetate was selected as the optimal solution to crude extraction of bacteriocin after comparing ammonium sulfate precipitation method and organic solvent extraction method, such as n‐butanol, n‐hexane, dichloromethane, trichloromethane, in which the diameter of the inhibition zones was above 28 mm. Results also showed the inhibition spectrum of the obtained bacteriocin had a broad spectrum of inhibition which could inhibit Gram‐positive, Gram‐negative, yeast. Especially, it could effectively inhibit *S. aureus* ATCC 25923, *Bacillus subtilis* CICC 10002, *Bacillus anthracis* CICC 20443, *E. coli* JM109 ATCC 67387, and *Salmonella* CMCC 541, and the zone diameter of inhibition has reached more than 28 mm. Moreover, it had a good thermal stability which antibacterial activity was retained 70.58% after treatment at 121°C for 30 min, and pH‐stability was between pH 2.0–9.0. These results suggested bacteriocin produced by *L. plantarum* zrx03 had potential application prospects in food preservation.

## Graphical abstract

This study investigated the crude extraction method and basic characteristics of a purified bacteriocin produced by Lactobacillus plantarum zrx03 isolated from infant's feces. This bacteriocin was considered to be a potential resource to develop natural and high‐efficiency preservatives, which could be well used in the food industry.

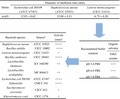

## 1 INTRODUCTION

The life quality of human being has also improved on the continuous advancement of technology. Consumers were increasingly concerned about the food safety, especially the preservation of food (Wayah & Philip, 2018). Most of the preservatives commonly used in the food industry were chemical preservatives containing organic acids and salts as the main components. Some food poisoning events and diseases were side effects caused by the abuse of chemical preservatives. Therefore, there was an urgent need to develop a new type of natural, efficient, and safe preservative to meet the needs of the market (Okuda, Tulini, Winkëlstroter, & Martinis, 2018; Wayah & Philip, 2018).

Lactic acid bacteria (LAB) were widely distributed among nature and were currently divided into at least 18 genera, with more than 200 species. Most of them were indispensable and important physiological functions of the human body. They were recognized as probiotics of the intestinal tract of human body and are applied to fermented foods (Azam et al., 2017; Ouiddir et al., 2019). Bacteriocin of LAB was a metabolite of lactic acid bacteria which was a kind of bacteriostatic protein synthesized by ribosomes (Wang et al., 2018). It can effectively inhibit the spoilage of food spoilage bacteria and a variety of pathogenic microorganisms (Goyal, Malik, & Pradhan, 2018; Kassaa et al., 2019; Mokoena, 2017), and bacteria were more immune to the produced bacteriocin (Milioni et al., 2015). It can be developed as a new preservative due to its natural nontoxicity and good selectivity (Hwanhlem, Ivanova, Biscola, Choiset, & Haertlé, 2017; Zhao et al., 2016). Nisin was the most representative LAB bacteriocin recognized by the public as a safe and harmless food additive (Ge, Teng, Wang, & Zhao,^2016^), but it had many shortcomings, for example, it had a narrow spectrum of inhibition which could be inhibited most Gram‐positive bacteria, but had no significantly affected on Gram‐negative bacteria, yeast, and mold. The limitations of nisin applications have made it a hot spot to find and research novel bacteriocin (Song, Zhu, & Gu, 2014; Yunyun, Zhaoxin, Jing, & Jie, 2015).

A variety of LAB species with different functional activities has been isolated from different sources. Goyal et al. (2018) obtained the bacteriocin‐producing *L. lactis* 63 from Indian dairy products, which was found to have a broad antimicrobial spectrum. Zhang et al. (2018) isolated *Lactobacillus plantarum* J23 from Chinese traditional fermented milk, the fermentation supernatant of which had a zone of inhibition diameter of more than 5 mm for *Listeria monocytogenes.* Hu et al. (2017) isolated *L. alimentarius* FM‐MM_4_ from Nanx Wudl, the fermentation supernatant had a zone of inhibition of 7.56 mm and 10.31 mm for *Escherichia coli* and *Staphylococcus aureus*, respectively. Al‐Seraih et al. (2017) isolated *Enterococcus faecalis* B3A‐B3B from infant feces, with activity against *L. monocytogenes*, *S. aureus*, methicillin‐resistant *Staphylococcus aureus* (MRSA), and *Clostridium perfringens*.

This experiment aims to isolate and identify a lactic acid bacteria with excellent antibacterial activity from the feces of infant and explore the crude extraction method and basic characteristics of a purified bacteriocin produced by *L. plantarum* zrx03 isolated from infant's feces to facilitate the research and development of *L. plantarum* bacteriocin‐related products.

## 2 MATERIALS AND METHODS

### 2.1 Isolation and screening of LABs

The fecal samples were the feces of the fourth day of the newborn, obtained from The Third Affiliated Hospital of Xinxiang Medical University. The sample was homogenized in 20 ml of sterile physiological saline solution. The homogenate was diluted with sterile physiological saline. Uniform distribution was spread on de Man‐Rogosa‐Sharpe (MRS) medium containing 0.1% calcium carbonate and incubated at 37°C for 36 hr. Colonies of clear areas were randomly selected and purified by repeated scribing on fresh MRS agar. Then, it was speculated that catalase‐negative and Gram‐positive colonies were mainly LABs and were stored in 25% (v/v) glycerol at −20°C.

### 2.2 Antimicrobial activity assay

The agar well assay (Hashim, Almasaudi, Azhar, Al Jaouni, & Harakeh, 2017) was used to detect antibacterial activity. Seven test LAB strains were isolated from infant's feces grown in MRS medium at 37°C for 18 hr. The cell‐free culture supernatant was obtained by centrifugation (10,000 *g*, 15 min, 4°C, Sigma), and then filtered through a 0.22 μm sterile filter. The indicator strains were *E. coli* JM109 (GenBank No. ATCC 67387), *S. aureus* (GenBank No. ATCC 25923), and *L. monocytogenes* (GenBank No. CTCC 21633) cultivated with different medium of Luria‐Bertani Medium (LB), Tryptic Soy Broth (TSB), and Brain Heart Infusion (BHI) to logarithmic phase. Plate was prepared by spreading the indicator bacterium (1.0 × 10^8^–1.5 × 10^8^ CFU/ml) onto their suitable agar medium, slotting wells about 6 mm. And then 100 μl LAB supernatant was added to the well and subsequently incubated under indicator bacterium suitable conditions. Zone of clearance around wells was observed to evaluate the sensitivity of the strains in question. The zone diameter of inhibition was measured using the cross method, and the well size was not counted.

### 2.3 Identification of the strain

The identification of the selected strain was confirmed on the basis of the 16S rRNA gene sequence. A DNA extraction kit (Takara) was used to extract and purify the genomic DNA. The 16S rRNA gene was amplified with the forward primer 27F 5′‐AGAGTTTGATCCTGGCTCAG‐3′ and a reverse primer 1492R 5′‐GGCTACCTTGTTACGACT‐3′ (Chen et al., 2014). PCRs were conducted with initial denaturation at 98°C for 3 min, followed by 30 cycles of denaturation at 98°C for 30 s, annealing at 54°C for 30 s, extending at 72°C for 3 min, and a final extension at 72°C for 10 min. PCR products were purified and sequenced by Sangong Biotech Co., Ltd. (Shanghai, China). Subsequently, the sequence was submitted to the GenBank database to conduct homologous analysis. MEGA 5.1 was used to construct the phylogenetic tree.

### 2.4 Determination of antimicrobial substance

The effects of other bacteriostatic substances such as organic acids and hydrogen peroxide produced by the strain during the fermentation process must be excluded. It was done by mixing 5 ml of the fermentation supernatant with catalase (Sigma) each at 5 mg/ml concentration on pH 7.0 and incubated at 37°C for 2 hr (Xu, Shi, Zhang, & Du, 2016). The effect of organic acids on the antimicrobial activity of the fermentation supernatant was tested to know whether the same acid concentration as the MRS medium with acetic acid, lactic acid, and hydrochloric acid had the sample antibacterial activity as the fermentation supernatant or not (Xu et al., 2018). The effect of enzymes on the antimicrobial activity of the fermentation supernatant was tested to know whether the inhibitory substance was proteinaceous in nature or not. It was done by mixing 5 ml of the fermentation supernatant with proteinase K, pepsin, trypsin, and papain (Sigma) each at 5 mg/ml concentration under its optimum pH conditions and incubated at 37°C for 2 hr (Wang et al., 2016). All the effect of the enzyme was terminated by boiling water bath for 3 min. The fermentation supernatant without any treatment was subjected to determine bacteriocin activity for the control. For all tests, the antimicrobial activity was determined by the agar well assay, using *E. coli* JM109 as an indicator strain.

### 2.5 Partial purification of bacteriocin

The antibacterial activity of crude bacteriocin extracted was as a basis of evaluation by comparing ammonium sulfate precipitation method and organic solvent extraction method, *E. coli* JM109 as indicator strain, to select the optimal conditions for crude extraction of bacteriocin.

The aliquot of fermentation supernatant was precipitated overnight with ammonium sulfate at 30°C to 40%–80% saturation levels at 4°C. The surface film and bottom precipitate were collected by centrifuging the mixture (10,000 *g*, 4°C, 30 min) and resuspended in phosphate buffer (0.01 M PBS, pH 5.0, 6.0, 7.0). This precipitate was subjected to dialysis through a membrane (Dialysis Membranes, MD25/8‐14 kDa, Solarbio) to remove salt.

The organic solvent, such as selected ethyl acetate, n‐butanol, n‐hexane, dichloromethane, trichloromethane, was mixed with the aliquot of fermentation supernatant, respectively, and extraction for 2 hr, and then the organic solvents were removed by rotary evaporation to obtain the crude extraction under its optimum temperature conditions. The crude extraction was reconstituted with 1/100 volume of PBS solution to different pH 5.0, 6.0, 7.0.

The antibacterial activity results of the fractions were expressed in arbitrary units (AU/ml). Antibacterial activity was measured using a serial double dilution method (Liu, Ren, Song, Wang, & Sun, 2015). Briefly, the bacteriocin supernatant was tested for antibacterial activity against *E. coli* JM109 after serial dilution twice with phosphate buffer (20 mM, pH 7.0). An AU has been defined as the reciprocal of the highest dilution, showing a clear zone of inhibition for the indicator strain. An arbitrary unit of bacteriocin was calculated according to the formula for 2*^n^* × 1,000/*x*, where *n* was the highest dilution that could significantly inhibit the indicator strain, and *x* was the amount of sample (μl) added to the well. The purification effect was evaluated by purification multiple and recovery. The following equations were used to calculate the purification folds and recovery yields to each purification step: purification folds = ratio of special activity; recovery = ratio of total viability. The protein concentration of all components was measured using the Pierce BCA (Dicaprylic Acid) Protein Assay Kit (Thermo Fisher Scientific).

### 2.6 Bacteriocin inhibition spectrum determination

The inhibition spectrum of the *L. plantarum* zrx03 crude bacteriocin extraction was determined by the agar well assay.

### 2.7 Sensitivity of bacteriocin

To evaluate thermal stability, 5 ml aliquots of bacteriocin were subjected to different heat treatments for 80, 90, 100, and 121°C, respectively, for 30 min with treated at room temperature as a control (Zhang et al., 2018). The effect of pH on the antimicrobial activity of the bacteriocin was tested by dissolving the bacteriocin sample of 5 ml of 1 M NaOH or 1 M HCl‐adjusted PBS solution at a pH range of 2.0–9.0 (in increments of one pH unit), untreated by acid or alkali as controls (Goyal et al., 2018). To evaluate enzyme stability, it was done by mixing 5 ml of the bacteriocin with pepsin, trypsin, papain, proteinase K, neutral protease, and alkaline proteinase (Sigma) each at 20 mg/ml concentration to keep the total volume and incubated at 37°C for 30 min (Hong et al., 2014). The enzymatic reactions were stopped by boiling for 3 min, and the antimicrobial activity was assessed by the agar well assay with untreated by enzyme solutions as controls. For all tests, the antimicrobial activity was determined by the agar well assay, using *E. coli* JM109 as an indicator strain.

### 2.8 Statistical analysis

All data were expressed the mean of three independent experiments and presented as mean values ± standard deviation (*SD*). All statistical analyses were performed with Origin 9.0 (Originlab).

## 3 RESULTS AND DISCUSSION

### 3.1 Isolation and identification of bacteriocin‐producing LABs

A number of 38 Gram‐positive and catalase‐negative LAB strains were isolated from infant's feces which colonies were moist and milky white, and the cells were mostly rod‐shaped under light microscope. The antibacterial test of *E. coli* JM109 as the target strain has a certain inhibitory effect. The cell‐free supernatants of seven strains showed strong inhibitory activity against *E. coli* JM109, *S. aureus,* and *L. monocytogenes* (Table 1). The fermentation supernatant of strain M31 had the strongest antibacterial activity against *E. coli*, and the zone diameter of the inhibition reached 13.83 ± 0.47 mm. The strain of the best bacteriostasis against *Staphylococcus aureus* and *L. monocytogenes* was strain zrx03, and the diameter of the inhibition zones reached 15.08 ± 0.31 mm and 6.75 ± 0.20 mm, respectively. The strain zrx03 was finally selected for further study as it exhibited the highest antimicrobial activity.

**Table 1:** The antimicrobial result of fermentation supernatant produced by different LAB strains

Stains	Diameter of inhibition zone (mm)		
	*Escherichia coli* JM109 (ATCC 67387)	*Staphylococcus aureus* (ATCC 25923)	*Listeria monocytogenes* (CICC 21633)
N1	12.08 ± 0.66	11.08 ± 0.51	5.08 ± 0.31
N2	12.50 ± 0.20	13.75 ± 0.41	1.08 ± 0.12
zrx03	12.83 ± 0.62	15.08 ± 0.31	6.75 ± 0.20
M13	12.50 ± 0.00	8.17 ± 0.42	1.25 ± 0.47
M21	12.17 ± 0.62	4.00 ± 0.20	2.00 ± 0.20
M31	13.83 ± 0.47	12.00 ± 0.61	2.92 ± 0.31
M37	12.00 ± 0.82	12.58 ± 0.31	1.92 ± 0.51

Note

Values represent means of triplicate ± standard deviation. The zone diameter of inhibition was measured using the cross method, and the pore size was not counted.

**Figure 1:**
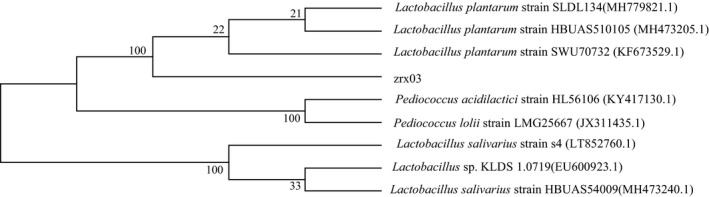
Phylogenetic tree of *L. plantarum* zrx03 based on 16S rRNA sequence

Therefore, the strain zrx03 was selected as the target strain and identified. The 16S rRNA gene was amplified, a 1,450 bp sequence was obtained and submitted to GenBank. Phylogenetic analysis showed 100% identity to the *Lactobacillus plantarum strain* (Figure 1). The zrx03 strain was preliminarily named as *L. plantarum* zrx03 (GenBank No. MN784485).

### 3.2 Determination of antimicrobial substance

**Figure 2:**
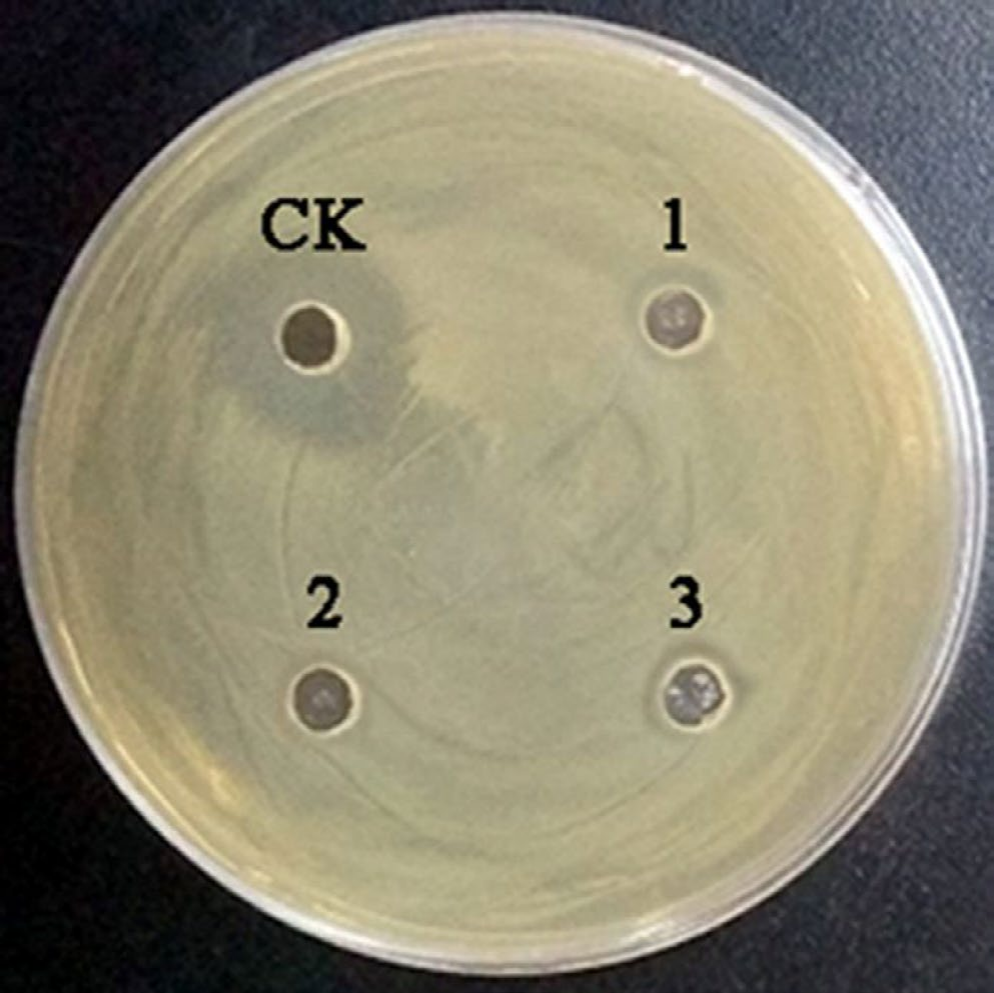
Antibacterial activity of *L. plantarum* zrx03 fermentation supernatant excluded organic acid influence. CK Fermentation supernatant control; 1 Acetic acid treated MRS medium; 2 Lactic acid treated MRS medium; 3 Hydrochloric acid treated MRS medium

**Figure 3:**
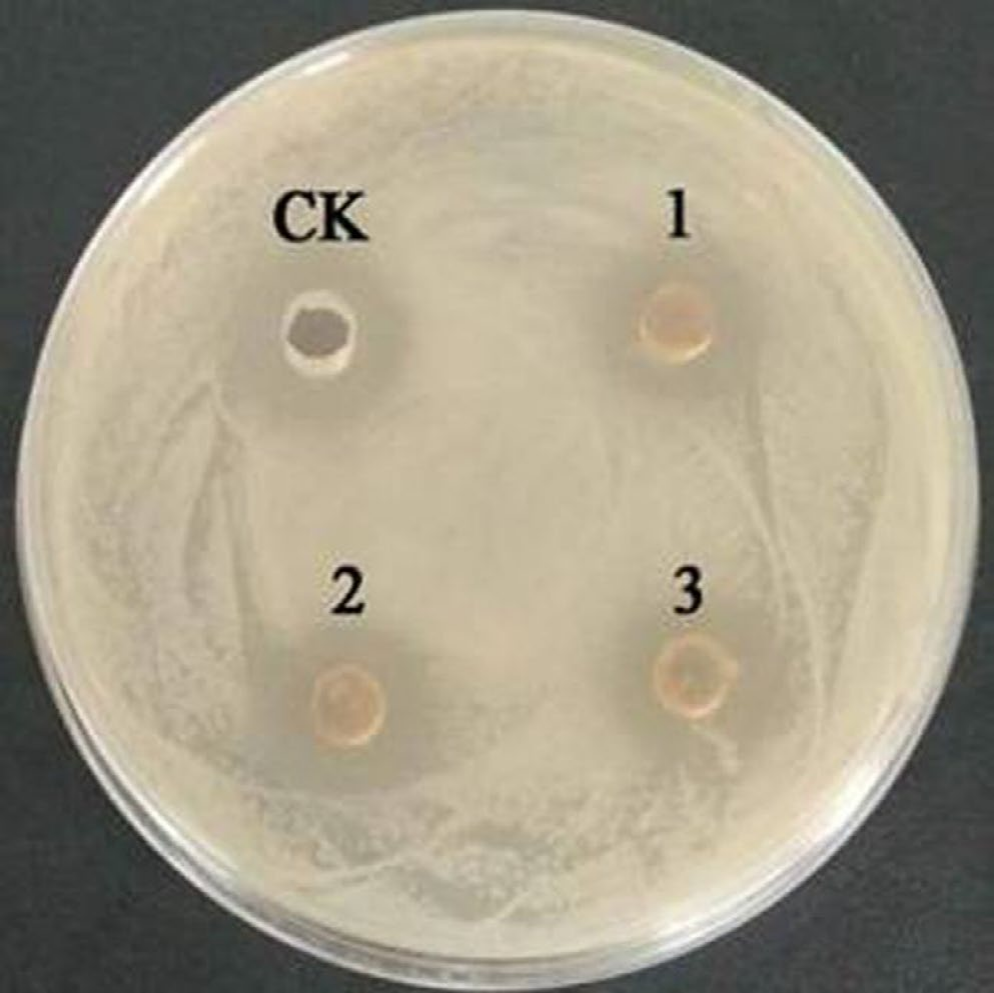
Antibacterial activity of *L. plantarum* zrx03 fermentation supernatant excluded hydrogen peroxide influence. CK Fermentation supernatant without catalase; 1, 2, 3 Fermentation supernatant treated with catalase

The organic acid‐treated MRS medium had no antibacterial activity, and the diameter of the inhibition zone of the fermentation supernatant after the hydrogen peroxide treatment was not changed compared with the control. The results indicated that neither organic acid nor catalase is the main substance for bacteriostasis (showed in Figures 2 and 3).

Based on the basic properties of the protein to determine whether an antimicrobial substance is a natural protein. Proteolytic enzymes such as pepsin, papain, trypsin, and proteinase K negatively affected the activity of antimicrobial substance of *L. plantarum* zrx03 (Table 2). The residual antibacterial activity of the fermentation supernatant after papain treatment was only 37.10%, and the antibacterial activity remained only 64.52% after trypsin treatment. The antimicrobial substance in the culture supernatant can thus be considered to be a bacteriocin since the protein properties of bacteriostatic substances had been observed on enzymatic action in other LAB bacteriocin studies (Furtado, Todorov, Landgraf, Destro, & Franco, 2014).

**Table 2:** Influence of different protease treatments on the antimicrobial activity of strain zrx03 fermentation supernatant

Enzyme	Pepsin	Trypsin	Papain	Proteinase K
Residual activity (%)	79.03 ± 2.28	64.52 ± 4.56	37.10 ± 0.00	77.42 ± 2.28

Note

Values represent means of triplicate ± standard deviation.

### 3.3 Preparation of bacteriocin crude extract

The diameter of the inhibition zone of the crude extract of bacteriocin obtained by ammonium sulfate precipitation was less than 18 mm (showed in Table 3). The antibacterial ability of the crude extract gradually increased in the increase in ammonium sulfate concentration. The diameter of the inhibition zone reached the maximum when 80% ammonium sulfate and pH 7 phosphate buffer solution was reconstituted.

**Table 3:** Antimicrobial activity of bacteriocin extracted by ammonium sulfate fractional precipitation

Reconstituted buffer solution	Different concentrations of ammonium sulfate				
	40%	50%	60%	70%	80%
pH 5.0 PBS	–	+	+	+	+
pH 6.0 PBS	–	+	+	+	++
pH 7.0 PBS	–	+	+	+	++

Note

Agar well assay for *L. plantarum* zrx03; ++++ = >28 mm; +++ = 18–28 mm; ++ = 8–18 mm; + = <8 mm; –, no inhibition.

N‐hexane, dichloromethane, and trichloromethane cannot be used as an extraction solvent for the crude extract of *L. plantarum* zrx03 bacteriocin since there was no antibacterial activity of the reconstituted hydrolysate after extraction (showed in Table 4). The diameter of the inhibition zone of the crude extract extracted with ethyl acetate and n‐butanol was above 18 mm, indicating that both of them had good extraction effects on bacteriocin. The crude bacteriocin extract obtained by using ethyl acetate as solvent and reconstituted in phosphate buffer solution to pH 7.0 had the best antimicrobial activity, which the diameter of the inhibition zone was above 28 mm.

**Table 4:** Antimicrobial activity of bacteriocin extracted with different organic solvents

Reconstituted buffer solution	Organic solvents				
	Ethyl acetate	N‐butanol	N‐hexane	Dichloromethane	Trichloromethane
pH 5.0 PBS	+++	+++	–	–	–
pH 6.0 PBS	+++	+++	–	–	–
pH 7.0 PBS	++++	+++	–	–	–

Note

Agar well assay for *L. plantarum* zrx03; ++++ = >28 mm; +++ = 18–28 mm; ++ = 8–18 mm; + = <8 mm; –, no inhibition.

It was found that the antibacterial activity of the crude extract prepared by extraction of ethyl acetate and n‐butanol was significantly higher than that of the ammonium sulfate fractionation after the comparison of ammonium sulfate precipitation and organic solvent extraction. Therefore, ethyl acetate extraction method and pH 7.0 phosphate buffer solution were selected as the preparation method of the crude extract of *L. plantarum* zrx03 bacteriocin.

The activity, yield, and purification fold of the bacteriocin, as obtained after each purification step, are presented in Table 5. The cell‐free supernatant from *L. plantarum* zrx03 was extracted by ethyl acetate, obtaining a crude extraction with a 23.86% recovery and a special activity of 5,775.89 AU/mg. Thus, *L. plantarum* zrx03 could be a multi‐bacteriocin producer with a significant exploitation and application value. This result was in accordance with that of other LABs, such as *L. plantarum* JY22 (Lv et al., 2017) and *Lactobacillus coryniformis* XN8 (Yi et al., 2016).

**Table 5:** Partial purification of bacteriocin

Purification stage	Volume (ml)	Activity (AU/ml)	Protein concentration (mg/ml)	Special activity (AU/mg)	Purification fold	Recovery (%)
Cell‐free supernatant	2,000	57.32	0.1390	412.37	1.00	100.00
Ethyl acetate extraction	20	1,367.73	0.2368	5,775.89	14.01	23.86

### 3.4 Antibacterial spectrum of bacteriocin

The inhibitory spectrum of *L. plantarum* zrx03 bacteriocin was assessed by the agar well assay against the stains listed in Table 6. The bacteriocin could strongly suppress common harmful bacteria and spoilage bacteria, such as *S. aureus*, *B. subtilis*, *B. anthracis*, *E. coli,* and *Salmonella*, which had an inhibition zone diameter above 28 mm. At the same time, it also had a certain inhibitory effect on *L. monocyto genes*. The bacteriocin also had the characteristics of most LAB bacteriocin, which has a certain inhibitory effect on the closely related *lactobacilli*. From Table 5, it was indicated the bacteriocin also had partial inhibition of the yeast. In a word, this bacteriocin could inhibit Gram‐positive, Gram‐negative, and yeast, so it was a broad‐spectrum bacteriocin.

**Table 6:** Antimicrobial spectrum of the bacteriocin produced by *L. plantarum* zrx03

Bacterial species	Source	Growth medium	Activity (mm)^a^
G^+^			
*Staphylococcus aureus*	ATCC 25923	TSB, 37°C	++++
*Bacillus subtilis*	CICC 10002	LB, 37°C	++++
*Listeria monocytogenes*	CICC 21633	BHI, 37°C	+
*Bacillus anthracis*	CICC 20443	LB, 28°C	++++
*Lactobacillus rhamnose*	KY 348290	MRS, 37°C	++
*Lactobacillus acidophilus*	MF 804413	MRS, 37°C	++
G^−^			
*Escherichia coli* JM109	ATCC 67387	LB, 37°C	++++
*Salmonella*	CMCC 541	LB, 37°C	++++
Saccharomyces			
*Saccharomyces cerevisiae*	CICC 012	YPD, 28°C	+
*Kluyveromyces cruzi*	CICC 1276	YPD, 28°C	+

Abbreviations: ATCC, American Type Culture Collection; BHI, Brain Heart Infusion; CICC, China Center of Industrial Culture Collection; CMCC, China Center of Medicine Culture Collection; LB, Luria‐Bertani Medium; MRS, de Man‐Rogosa‐Sharpe Medium; TSB, Tryptic Soy Broth medium; YPD, Yeast Extract Peptone Dextrose Medium.

a Agar well assay for *L. plantarum* zrx03; ++++ = >28 mm; +++ = 18–28 mm; ++ = 8–18 mm; + = <8 mm; –, no inhibition.

### 3.5 Sensitivity of bacteriocin to temperature, pH, and protease

The effect of enzymes, temperature, and pH on the antimicrobial activity of *L. plantarum* zrx03 was presented in Table 7. The antibacterial ability of *L. plantarum* zrx03 bacteriocin decreased from the increase in temperature, and the low temperature had a little effect on its antibacterial activity at 4°C. From the Table 6, it was showed that the bacteriocin had good thermal stability which its antibacterial activity still maintained above 90% after treatment of 40°C and 60°C for 30 min, even the activity could still reach 71.81% after treatment at 121°C for 30 min. The antibacterial activity of bacteriocin decreased slightly from the increase in pH from 2.0 to 9.0, which the antibacterial activity of 85.84% was maintained at pH 9.0. It could be seen from Table 6 that the bacteriocin produced by *L. plantarum* zrx03 had different degrees of antibacterial activity under the action of different proteases, among which the antibacterial activity of bacteriocin was remained 70.21% when treated by proteinase K.

**Table 7:** Sensitivity of bacteriocin to temperature, pH, and protease

Temperature	Residual activity (%)	pH	Residual activity (%)	Protease	Residual activity (%)
4°C	98.45 ± 0.82	Control (1.91)	100.0 ± 1.14	Control	100 ± 1.82
Control (Room temperature)	100 ± 0.72	3	97.36 ± 2.21	Pepsin	85.62 ± 0.33
40°C	94.58 ± 2.13	4	96.21 ± 2.24	Trypsin	77.40 ± 0.71
60°C	92.27 ± 0.27	5	92.18 ± 2.34	Papain	75.08 ± 1.86
80°C	87.45 ± 0.81	6	91.14 ± 0.89	Proteinase k	70.21 ± 1.02
100°C	80.69 ± 2.13	7	91.14 ± 2.79	Neutral protease	78.79 ± 0.82
121°C	71.81 ± 0.82	8	88.134 ± 1.6	Alkaline proteinase	78.55 ± 1.02
		9	85.84 ± 0.95		

Note

Values represent means of triplicate ± standard deviation.

## 4 CONCLUSIONS

In this study, a strain of LAB with strong antibacterial activity was isolated from infant's feces. After identification by 16S rRNA and phylogenetic tree analysis, it was named as *L. plantarum* zrx03. The fermentation supernatant of this strain showed good bacteriostasis after excluding the interference with organic acid and hydrogen peroxide. Comparing the antibacterial ability of the crude extract obtained by ammonium sulfate precipitation and organic solvent extraction, the ethyl acetate extraction method was the optimal solution, which the crude extract obtained by reconstitution with pH 7.0 phosphate buffer solution had the strongest antibacterial ability. The cell‐free supernatant from *L. plantarum* zrx03 was extracted by ethyl acetate, obtaining a crude extraction with a 23.86% recovery and a special activity of 5,775.89 AU/mg. Most Gram‐positive and negative pathogens such as *Staphylococcus aureus*, *Bacillus subtilis*, *Bacillus anthracis*, *E. coli* JM109, and *Salmonella* could be inhibited by this bacteriocin, some closely related LABs could be also inhibited. Moreover, the purified bacteriocin had to heat resistance and acid and alkali resistance. 70.58% of the antibacterial activity was retained after treatment for 121°C for 30 min, 87.65% of bacteriostatic activity was maintained after treatment of pH 9.0 for 2 hr, and it had a certain sensitivity to trypsin, papin, and proteinase K.

This study explored a better crude extraction method of *L. plantarum* zrx03 bacteriocin and demonstrated this bacteriocin had a strong antibacterial activity and was a broad‐spectrum bacteriostatic substance, which had a thermal stability and pH‐stability. Therefore, it was considered to be a potential resource to develop natural and high‐efficiency preservatives, which can be well used in the food industry.

## CONFLICT OF INTEREST

All authors declare no conflict of interest.

## ETHICAL STATEMENT

This article does not contain any studies with human participants or animals performed by any of the authors. Informed consent was obtained from all individual participants included in the study.
